# Rapamycin retards growth and causes marked alterations in the growth plate of young rats

**DOI:** 10.1007/s00467-007-0456-8

**Published:** 2007-07-01

**Authors:** Oscar Alvarez-Garcia, Eduardo Carbajo-Pérez, Enrique Garcia, Helena Gil, Ines Molinos, Julian Rodriguez, Flor A. Ordoñez, Fernando Santos

**Affiliations:** 1 Universidad de Oviedo, Oviedo, Spain; 2 Instituto Universitario de Oncologia del Principado de Asturias, Oviedo, Spain; 3 Hospital Alvarez Buylla, Mieres, Spain; 4 Hospital Universitario Central de Asturias, Oviedo, Spain; 5 Departamento de Morfologia y Biologia Celular, Facultad de Medicina, c/Julian Claveria 6, 33006 Oviedo, Spain

**Keywords:** Sirolimus, Rapamycin, Transplantation, Growth, Growth plate, Rat

## Abstract

Rapamycin is a potent immunosuppressant with antitumoral properties widely used in the field of renal transplantation. To test the hypothesis that the antiproliferative and antiangiogenic activity of rapamycin interferes with the normal structure and function of growth plate and impairs longitudinal growth, 4-week-old male rats (*n* = 10/group) receiving 2 mg/kg per day of intraperitoneal rapamycin (RAPA) or vehicle (C) for 14 days were compared. Rapamycin markedly decreased bone longitudinal growth rate (94 ± 3 vs. 182 ± 3 μm/day), body weight gain (60.2 ± 1.4 vs. 113.6 ± 1.9 g), food intake (227.8 ± 2.6 vs. 287.5 ± 3.4 g), and food efficiency (0.26 ± 0.00 vs. 0.40 ± 0.01 g/g). Signs of altered cartilage formation such as reduced chondrocyte proliferation (bromodeoxiuridine-labeled cells 32.9 ± 1.4 vs. 45.2 ± 1.1%), disturbed maturation and hypertrophy (height of terminal chondrocytes 26 ± 0 vs. 29 ± 0 μm), and decreased cartilage resorption (18.7 ± 0.5 vs. 31.0 ± 0.8 tartrate-resistant phosphatase alkaline reactive cells per 100 terminal chondrocytes), together with morphological evidence of altered vascular invasion, were seen in the growth plate of RAPA animals. This study indicates that rapamycin can severely impair body growth in fast-growing rats and distort growth-plate structure and dynamics. These undesirable effects must be kept in mind when rapamycin is administered to children.

## Introduction

Rapamycin has raised great interest in the field of renal transplantation in recent years. Early studies were performed in adults in which rapamycin was used as a rescue agent for acute and chronic graft rejection as well as for primary immunosuppression [1]. Rapamycin-based regimens have been reported to provide effective rejection prophylaxis without the risks of hypertension and decline in renal function associated with calcineurin inhibitors [2]. Most interestingly, rapamycin treatment in combination with other immunosuppressants has been reported to improve renal function [3, 4] and to decrease the elevated risk of developing cancer in renal transplanted patients [5].

At present, there is very little information on the use of rapamycin in pediatric solid organ transplantation [1, 6, 7]. The effects of rapamycin on longitudinal growth have not been assessed in pediatric clinic trials. Rapamycin has been reported to reduce cellular proliferation in several in vitro models [8] and to inhibit vascular endothelial growth factor (VEGF) synthesis in animals [9]. Chondrocyte proliferation and vascular invasion of the epiphyseal growth plate are two milestones of the endochondral ossification process. Therefore, rapamycin has the potential to alter growth-plate function and impair longitudinal bone growth. In animal studies, rapamycin administration has been shown to reduce weight gain [10, 11]. In addition, decreased longitudinal growth rate has been reported in adult rats given rapamycin [12].

Based on the above rationale, the study presented here explored the hypothesis that rapamycin treatment might adversely influence growth, and growth-plate structure and dynamics of fast-growing young individuals.

## Animals and methods

Male Sprague-Dawley rats aged 4 weeks and weighing 70 ± 5 g were housed in individual cages under controlled conditions of light (12 h light/dark cycle) and temperature (21–23°C). All animals received standard rat chow (A03, Panlab, Barcelona, Spain) and tap water ad libitum. The study complied with current legislation on animal experiments in the European Union and was approved by the Ethical Committee on Animal Research of our institution.

### Experimental protocol and sample collection

After 4 days of acclimation, animals were classified in two groups of ten individuals each: control (C) and rapamycin-treated (RAPA) rats. From day 0 to day 13 of the protocol, 2 mg/kg per day of rapamycin, purchased from LC Labs (LC Laboratories, Woburn, MA, USA), were administered intraperitoneally at 10 a.m. to the RAPA group. C animals received intraperitoneal injections of vehicle (5% dimethylsulfoxide; DMSO; Sigma, St. Louis, MO, USA). Animals were sacrificed under anesthesia on day 14 of the protocol approximately at the time of daily rapamycin injection. Three days before sacrifice, each animal received 30 mg/kg of calcein (Sigma, St. Louis, MO, USA) by intraperitoneal route. Bromodeoxyuridine (BrdU; 100 mg/kg; Sigma) was injected intraperitoneally 1, 9 and 17 h before death. At sacrifice, blood samples were collected and stored at −20C° until measurement of rapamycin concentrations. Tibiae were removed and the proximal ends fixed and processed for methyl methacrylate embedding, as previously described [13]. Right tibiae were fixed in 40% ethanol for analysis of calcein labeling and BrdU immunohistochemistry. Left tibiae were fixed in 4% neutral formalin at 4°C and used for other morphometric, cytochemical, and immunocytochemical studies.

### Rapamycin blood concentration

Trough concentrations of rapamycin were measured in whole blood using an Abbott IMx analyzer using the IMx sirolimus MEIA kit (Abbott Laboratories, Abbott Park, IL, USA) following manufacturer’s instructions.

### Growth and nutrition

Food intake and body weight were measured daily using an electronic balance (Ohaus GT 2100, Florham Park, NJ, USA). Nose to tail-tip length was measured under anesthesia on day 14. Food efficiency was calculated as the ratio between weight gained and food consumed (g/g) by each animal between days 0 and 13 of the protocol. Longitudinal growth rate was measured in 10-μm-thick frontal sections of the proximal end of tibiae obtained using a rotary microtome (HM355S, Microm, Barcelona, Spain) fitted with tungsten carbide blades. Sections were examined under an Olympus incident light fluorescence microscope (Olympus BX41, Olympus Optical España, Barcelona, Spain) coupled to a digital camera (Olympus DP11, Olympus Optical España, Barcelona, Spain) to detect calcein label. Images were captured and the distance between the chondro-osseous junction, and the calcein label was measured using an image analysis system (Scion Image, Scion Corporation, Frederick, MD, USA). The average value of these measurements divided by three was considered as the daily longitudinal bone growth during the last 3 days of either rapamycin or vehicle administration.

### Histology and histomorphometry

Frontal sections (5-μm thick) of proximal tibiae fixed in formalin were stained by the following methods: alcian blue/safranine for morphometric analysis, Von Kossa staining for mineralization analysis, tartrate-resistant acid phosphatase (TRAP) stain for osteoclast identification, periodic acid-Schiff (PAS) reaction to stain glycogen deposits, and picrosirius red/alcian blue/hematoxylin for analysis of bone and cartilage extracellular matrix. Heights of growth cartilage and its hypertrophic zone were identified following morphologic criteria and measured at regular intervals using an image analysis system described elsewhere [13]. Height of the three most distal hypertrophic chondrocytes was measured in alternate columns using the same system. The number of TRAP-positive cells at the vascular invasion front was measured in an area extending 50 μm from the distal end of growth cartilage into the primary spongiosa in two slides per animal. The results were expressed as the number of positive cells per 100 terminal chondrocytes.

### Immunohistochemical analysis of proliferating chondrocytes

Immunodetection of BrdU-labeled cells was carried out as previously described [13]. Briefly, after deplastination and rehydration, frontal 5-μm-thick sections were rinsed in 0.1 M Tris-hydrochloric buffer and treated with trypsin (1 mg/ml, 0.1% CaCl_2_; 60 min, 37°C). Endogenous peroxidase activity was inactivated by 30-min treatment in 3% hydrogen peroxide, and horse serum (1:5, 75 min; Sigma, St. Louis, MO, USA) was used to block unspecific reactions. Then, samples were incubated for 48 h (4°C in a moist chamber) with monoclonal antibody to BrdU (1:20; Dako Diagnosticos SA, Barcelona, Spain) followed by reaction with anti-mouse secondary conjugated antibody (EnVision, Dako Diagnosticos SA). The final reaction product was revealed with diaminobenzidine, and sections were lightly counterstained with alcian blue. Two sections were analyzed per animal, and the proliferating activity was expressed as the number of positive cells per 100 cells in the proliferative zone, defined for this purpose as the band of tissue comprised between the resting zone and a line traced by the most distal BrdU-labeled cells.

### Immunohistochemical analysis for VEGF

VEGF immunohistochemical staining was performed in 5-μm-thick sections of the proximal end of tibiae fixed in formalin. After deplastination in acetone and rehydration, all sections were treated with hydrogen peroxide and goat serum as described above. Then, sections were incubated overnight at 4°C with a 6/100 solution of polyclonal anti-VEGF antibody (Neo Markers, Westinghouse, CA, USA). After 30 min incubation with anti-rabbit secondary conjugated antibody (EnVision; Dako Diagnosticos), the final reaction product was revealed with diaminobenzidine and methyl green was used as counterstaining.

### Statistical analysis

Values are given as mean ± standard error of the mean (SEM). Comparisons among groups were carried out by Student’s* t* test. A* P* value ≤0.05 was considered significant. All data sets were analyzed using SPSS 11.0 software package (SPSS Inc., Chicago, IL, USA).

## Results

**Fig. 1:**
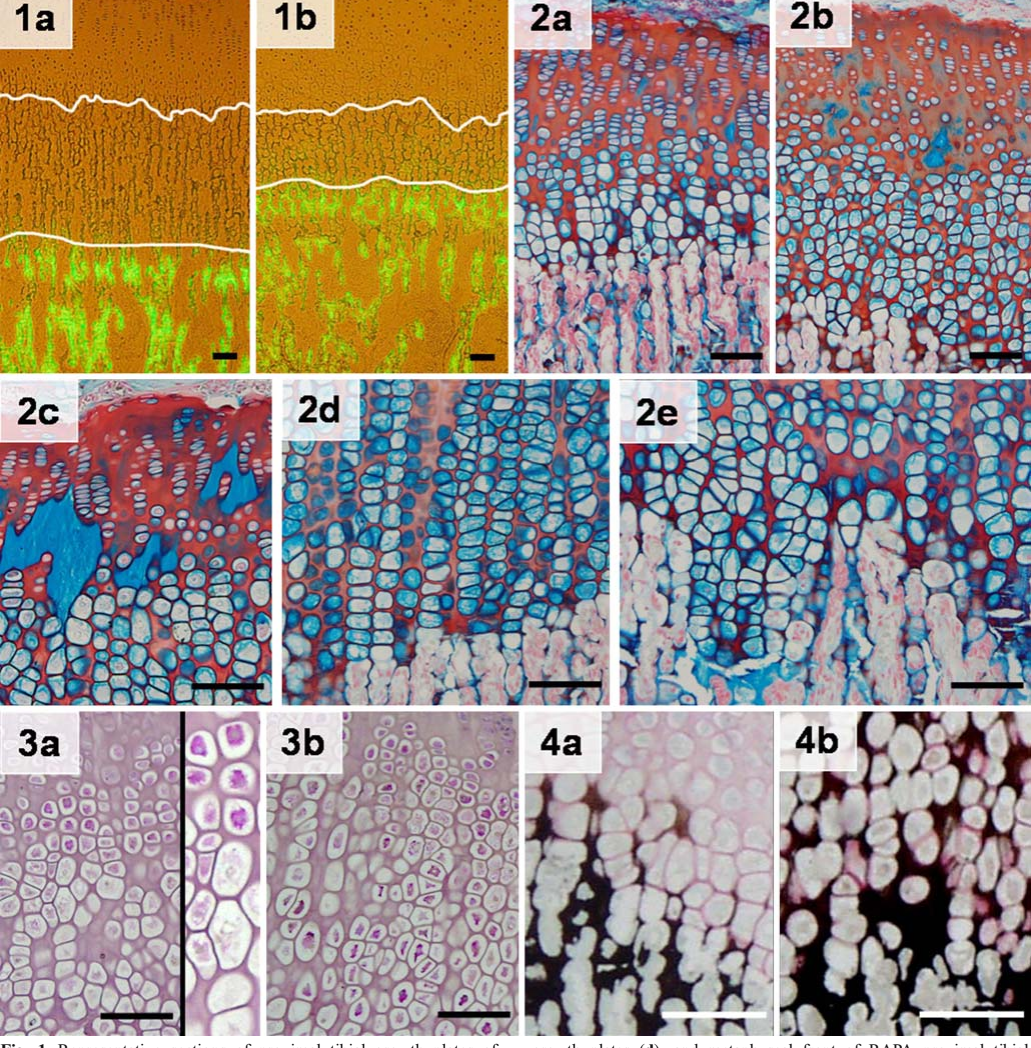
Representative sections of proximal tibial growth plates of control (C; **a**) and rapamycin-treated (RAPA; **b**) rats. The mean distance between the metaphyseal end of the growth plate and the fluorescent calcein front indicates longitudinal bone growth rate during the last 3 days of the study.* Magnification bars*: 100 μm Fig. 2. Representative sections of proximal tibial growth plates of control (C; **a**) and rapamycin-treated (RAPA; **b**–**e**) animals stained with alcian blue/safranine. Details of proliferative zone of RAPA proximal tibial growth plates (**c**), hypertrophic zone of RAPA proximal tibial growth plates (**d**), and metaphyseal front of RAPA proximal tibial growth plates (**e**).* Magnification bars*: 100 μm Fig. 3. Periodic acid-Schiff (PAS) reaction in the proximal tibial growth plate of control (C; **a**) and rapamycin-treated (RAPA; **b**) animals. Cytoplasmic glycogen deposits in control rats are shown at greater augmentation.* Magnification bars*: 100 μm Fig. 4. Von-Kossa staining showing matrix mineralization of proximal tibial growth plates of control (C; **a**) and rapamycin-treated (RAPA; **b**) animals.* Magnification bars* 100 μm

Rapamycin trough blood levels in the RAPA group were 22.3 ± 2.1 ng/ml. As shown in Table 1, these animals exhibited marked growth impairment, as demonstrated by reductions in weight gain, nose to tail-tip length, and longitudinal bone growth rate when compared with control animals. Significant decreases in food intake and food efficiency were also observed in RAPA animals when compared with C animals. Representative images of tibial growth-plate sections illustrating the differences in longitudinal bone growth rate are shown in Fig. 1.

**Table 1:** Growth and food intake data [mean ± standard error of the mean (SEM)] of control rats (C) and rats treated with rapamycin (RAPA)

	C	RAPA
Weight gain (g)	113.6 ± 1.9	60.2 ± 1.4*
Length (cm)	35.1 ± 0.2	32.4 ± 0.1*
Longitudinal growth rate (μm/day)	549 ± 13	279 ± 17*
Foot intake (g)	287.5 ± 3.4	227.8 ± 2.6*
Food efficiency (g/g)	0.40 ± 0.01	0.26 ± 0.00*

Food efficiency was calculated as grams of gained weight divided by grams of consumed food

*P < 0.05

### Histology and histomorphometry of the growth cartilage

Heights of both the growth cartilage and its hypertrophic zone greatly varied in RAPA animals, sometimes being greater than in C animals (Fig. 2a,b). The histomorphometric analysis did not demonstrate significant differences in either the height of epiphyseal cartilage (C: 493 ± 32 μm; RAPA: 629 ± 47 μm) or the height of the hypertrophic zone (C: 347 ± 28 μm; RAPA: 437 ± 38 μm).

Even in a context of evident morphological variability, some histological features were consistently present in most samples from RAPA animals (Fig. 2b–e). The proliferative zone was frequently disorganized, with loss of the normal columnar pattern. Acellular regions of fibrinous material markedly stained with alcian blue were often found in this zone (Fig. 2c). The transition between proliferative and hypertrophic zones was sometimes ill defined; others were quite abrupt (Fig. 2b–c). Groups of flattened hypertrophic chondrocytes, with their long axis oriented perpendicular to the longitudinal axis of the bone, were found near the distal end of cartilage (Fig. 2d). Morphometric analysis revealed that the mean height of terminal chondrocytes was significantly lower in RAPA (26 ± 0 μm) than in C animals (29 ± 0 μm). The chondro-osseous junction in RAPA samples was not as neat and regular as in C animals, with zones distorted by clusters of hypertrophic chondrocytes extending into the depth of the primary spongiosa (Fig. 2e). Isolated chondrocytes were occasionally seen within the metaphyseal bone.

In C animals, PAS-positive cytoplasmic granules were clearly seen in chondrocytes of the upper hypertrophic zone (Fig. 3a), and marked cells were gradually disappearing toward the vascular invasion front. In RAPA samples, chondrocytes containing PAS-positive material were seen in a wider area, including most of the hypertrophic zone, and strongly stained chondrocytes were identifiable near the distal end of the growth cartilage (Fig. 3b). Von-Kossa staining (Fig. 4) revealed that mineralization of cartilage matrix in control animals (Fig. 4a) was mostly confined to longitudinal septa flanked by the last two or three chondrocytes of adjacent columns. In RAPA samples (Fig. 4b), a wider band of mineralized cartilage matrix was seen, mineralization affecting not only to longitudinal septa but also to transversal septa. In this group, chondrocytes were often seen immersed in a mass of mineralized matrix.

### Cellular proliferation in growth cartilage

**Fig. 5:**
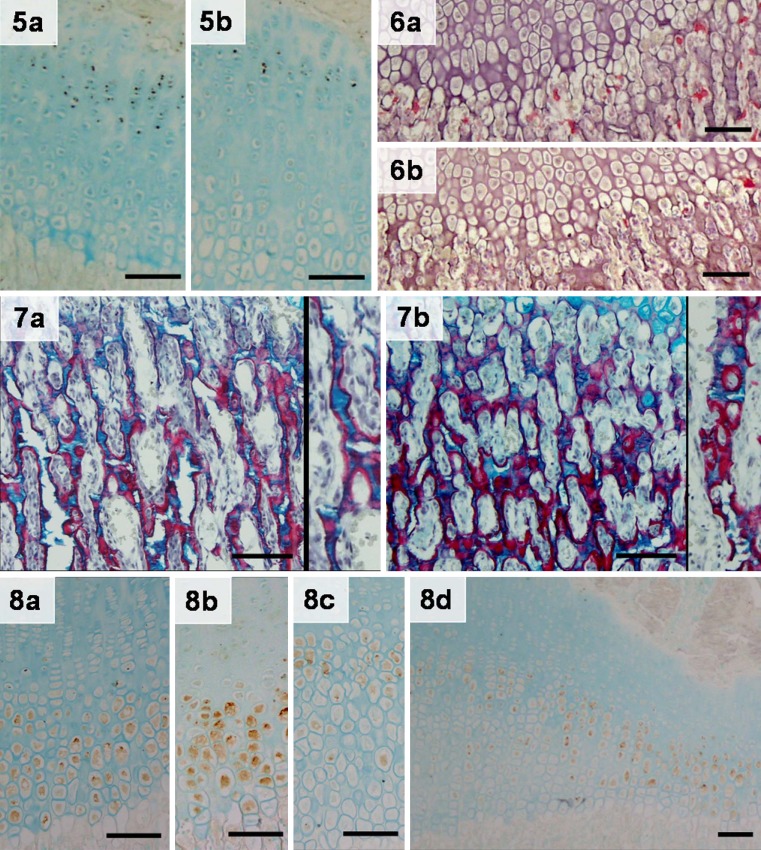
Bromodeoxyuridine (BrdU)-labeled nuclei in the proximal tibial growth plate of control (C; **a**) and rapamycin-treated (RAPA; **b**) animals counterstained with alcian blue.* Magnification bars*: 100 μm Fig. 6. Tartrate-resistant acid phosphatase (TRAP) stain in proximal tibial growth plates of control (C; **a**) and rapamycin-treated (RAPA; **b**) animals.* Magnification bars*: 100 μm Fig. 7. Picrosirius red/alcian blue/hematoxylin staining of proximal tibial growth plates of control (C; **a**) and rapamycin-treated (RAPA; **b**) animals with details of the structure of the primary trabeculae.* Magnification bars*: 100 μm Fig. 8. Vascular endothelial growth factor (VEGF) immunohistochemical stain counterstained with methyl green in proximal tibial growth plates of control (C; **a**) and rapamycin-treated (RAPA; **b**, **c**, **d**) animals.* Magnification bars*: 100 μm

BrdU-labeled nuclei were easily identified throughout the proliferative zone in samples from both groups (Fig. 5). The percentage of labeled nuclei in the proliferative zone was significantly higher in C (45.2 ± 1.1%) (Fig. 5a) than in RAPA (32.9 ± 1.4%) samples (Fig. 5b).

### Vascular invasion front and primary spongiosa

Dispersed TRAP-positive elements (chondroclasts/osteoclasts) were found in septa of the primary spongiosa and along the vascular invasion front in the two groups of rats (Fig. 6). Direct examination under the microscope showed that TRAP-positive elements at the chondro-osseous junction had a somewhat regular distribution in C animals (Fig. 6a)—approximately one every three terminal chondrocytes. In the same area of RAPA samples (Fig. 6b), fewer and irregularly distributed TRAP-reactive cells were seen. The number of TRAP-reactive cells per 100 terminal chondrocytes in RAPA samples (18.7 ± 0.5) was less than that of controls (31.0 ± 0.8).

Picrosirius red/alcian blue staining highlighted morphological and structural differences in the primary spongiosa of both groups (Fig. 7). Thin longitudinal septa, parallel to the long axis of bone, defined the primary spongiosa in control animals (Fig. 7a). These longitudinal trabeculae, built on a nucleus of cartilage matrix-blue–covered by a thin layer of bone matrix-red (insert in Fig. 7a)–marked the path of invading capillary sprouts. The longitudinal arrangement of septa and vessels was distorted in RAPA samples (Fig. 7b) by the presence of transverse unresorbed septa made of cartilage and/or bone matrix. As opposed to the slender structure of primary trabeculae found in control animals, thicker trabeculae, including empty rings of cartilage or bone matrix, were often seen in the primary spongiosa of treated animals (insert in Fig. 7b).

### Immunohistochemistry for VEGF

VEGF immunohistochemical signal was intracellular and localized within the cytoplasm of hypertrophic chondrocytes in the control group (Fig. 8a). In RAPA animals, no uniform pattern for VEGF immunostaining was seen (Fig. 8b–d). In some cases, VEGF immunostaining was indistinguishable from that seen in controls (Fig. 8b). In other samples, VEGF signal was found irregularly dispersed in the hypertrophic zone, and terminal chondrocytes were rarely immunopositive (Fig. 8c). In some sections, both patterns coexisted in the same sample, particularly in those samples with narrow and widened cartilage zones (Fig. 8d). A regular staining of the hypertrophic zone was generally found in nonexpanded areas of the growth cartilage whereas scanty and dispersed labeled cells were seen in areas of expanded hypertrophic zone.

## Discussion

The study presented here shows that administration of rapamycin to fast-growing rats impairs longitudinal growth and induces marked alterations in growth-plate structure. In agreement with previous studies in adult animals [10, 11, 14, 15], rapamycin treatment was followed by a significant reduction of body-weight gain and food intake. Interestingly, the lower food efficiency of animals given rapamycin compared with that of controls clearly shows that reduction of body-weight gain was not just caused by food intake reduction but points to a direct negative effect of rapamycin on weight gain. Oral gavage has been reported to significantly decrease both food intake [16] and rapamycin biodisponibility [17]. To avoid these adverse effects, we injected the drug intraperitoneally, unlike former reports [10, 15] in which rapamycin was given by oral gavage. Although trough rapamycin levels in the treated animals of our study were slightly above those previously reported to induce immunosuppression in rats [10], no evidence of toxicity was noted. Additional studies will be of interest to identify the critical ages and doses at which rapamycin inhibits normal growth.

The marked decrease of longitudinal growth rate in rapamycin-treated young animals was a relevant finding of this study. Romero et al. [12], in the only study to date reporting the effect of rapamycin on longitudinal growth, stated that the reduction of growth velocity (about 30%) found in their experimental model in adult rats might be of little importance in adults but would acquire special relevance in young individuals. This is fully confirmed in the present report. We found a longitudinal growth rate reduction of approximately 50% in young animals treated with rapamycin in comparison with control animals. Further experiments including pair-fed animals will be needed to clarify the role played by rapamycin-induced food intake reduction in this remarkable fall of growth rate. However, on the base of our experience with food restriction in several experimental models using rats of similar age, it can be pointed out that the severe growth retardation found in the rats treated with rapamycin was not mainly due to food intake reduction. We have previously shown [18] that a 37% food intake restriction decreases daily growth rate approximately 13%, whereas the 20% food intake reduction of the RAPA group reduced growth velocity to 50%. Further to this, the mild growth retardation found in the above-mentioned food-restricted animals was never accompanied by the pronounced alterations of growth-plate morphology found in the rapamycin-treated animals.

The effects of rapamycin on bone metabolism have been addressed by several studies in recent years [12, 19, 20], but its effects on the growth plate are analyzed here for the first time. Taking together data from the growth cartilage and the adjacent primary spongiosa, our study indicates that both the production and degradation of epiphyseal cartilage were adversely affected by rapamycin treatment.

As shown by the immunostaining of BrdU-labeled nuclei, proliferation of growth-plate chondrocytes was markedly diminished by rapamycin treatment. This, indeed, contributed to a decline in the production of growth cartilage. The reduction of the proliferative activity of chondrocytes may likely be related to the antiproliferative action of rapamycin. Mammalian target of rapamycin (mTOR) has been described as a downstream effector of insulin-like growth factor type I (IGF-I) in various experimental studies [21–24]. Therefore, it can be hypothesized that blockade of the IGF-I/mTOR metabolic route by rapamycin would partly inhibit IGF-I stimulus of cellular proliferation, hence decreasing chondrocyte proliferation rate.

Several findings of our study, such as the disorganization of both proliferative and maturative zones, the abnormal presence of glycogen deposits in distal hypertrophic chondrocytes, and the flattening of terminal hypertrophic chondrocytes, support the notion that rapamycin altered the normal process of maturation and hypertrophy of chondrocytes. The decreased height of terminal hypertrophic chondrocytes may play an important role in the decreased growth rate of the animals treated with rapamycin because chondrocyte enlargement supports nearly 60% of longitudinal bone growth in young rats [25], and height of terminal chondrocytes and longitudinal growth rate have been shown to be positively correlated [18].

Morphological distortion of the maturation zone and presence of PAS-positive glycogen granules in distal hypertrophic chondrocytes have been previously interpreted as a disruption of the orderly process of maturation of chondrocytes in a model of parathyroid-hormone-related peptide (PTHrP)-depleted mice [26]. Therefore, our results encourage the further study of rapamycin interaction with the Indian hedgehog (Ihh)/PTHrP feedback loop [27], so far not explored.

The presence of acellular regions of fibrinous material in the proliferative zone and the altered mineralization of transverse septa might well stand for extracellular matrix alterations. Beyond providing support for growth-plate chondrocytes, the extracellular matrix is involved in the control of growth factor diffusion [27] and is linked to cartilage mineralization. The matrix structure varies in transverse and longitudinal cartilage septa [28] and, worthy of note, matrix mineralization does occur in longitudinal septa of the lower hypertrophic zone, whereas transverse septa are rarely mineralized [29]. Recent studies indicate that perivascular cells degrade unmineralized matrix and transverse septa, whereas osteoclasts are involved in degradation of longitudinal septa and mineralized cartilage [30]. Our data support that degradation of cartilage matrix by both the action of osteoclasts and that of invading capillary sprouts is compromised in rapamycin-treated animals. On the one hand, rapamycin treatment yielded a significant reduction in the number of TRAP-positive cells at the metaphyseal front. This agrees with the decreased number of osteoclasts and osteoclast activity recently reported in adult rats receiving everolimus [31]. On the other hand, the arrangement of capillary sprouts among slender longitudinal septa in the primary spongiosa was clearly distorted after rapamycin treatment, with many vascular channels running parallel to the chondro-osseous junction. This could be the result of certain matrix refractoriness to resorption stimuli and/or the alteration of the signaling pathways controlling vascular invasion.

Growth-plate vascularization is a key step required for degradation of cartilage and bone formation [32, 33]. It is of note that hypertrophic chondrocytes play a significant role in the process of vascular invasion by producing angiogenic factors, such as VEGF [33] or fibroblast growth factor (FGF) [34], and angiostatic molecules, such as chondromodulin [35]. The premise that rapamycin inhibits VEGF synthesis [9] led us to analyze the expression of VEGF by growth-plate chondrocytes. As opposed to the rather homogeneous VEGF immunostaining of the hypertrophic zone both in control rats and in nonexpanded areas of growth cartilage from treated animals, scanty and dispersed labeled cells were seen in areas of widened hypertrophic zone from RAPA samples. This is consistent with the increased width of hypertrophic zone and suppression of vascular invasion found after inactivation of VEGF in mice [33] and supports a heterogeneous effect of rapamycin in the growth plate, with somewhat focal alterations.

Removal of terminal chondrocytes by programmed cell death is of undeniable relevance for the homeostasis of the growth plate. However, conceptual and technical approaches to quantify this phenomenon are really controversial [36, 37]. No attempt was made here to evaluate programmed cell death, but the presence of well-preserved, isolated chondrocytes in the primary spongiosa of RAPA samples strongly suggests that chondrocyte removal from the growth cartilage was also impaired by rapamycin treatment.

In summary, our study shows that administration of rapamycin to young, fast-growing individuals severely impairs growth and the structure and dynamics of long-bone growth plate. These findings open the way to further studies aimed at exploring the pathogenic mechanisms of these effects and warn about the extensive use of rapamycin in transplanted children until studies analyzing the impact of rapamycin treatment on growth in the clinical setting are accomplished.
